# Emigrants: Whence They Come and Where They Go

**Published:** 1894-11-24

**Authors:** 


### Studies in Applied Sociology.

### EMIGRANTS: WHENCE THEY COME AND WHERE THEY GO.

When we speak of the American nation, we do not mean the same thing as when we speak of the English, the French, the German nation. Till this century, indeed, the Anglo-Saxon element predominated, except in the South, where the descendants of the original French settlers maintained their language and customs. The United States of America, when they were first so constituted, formed fundamentally a branch of the English race, hating England in politics, perhaps, and affecting, as it does to-day, a superficial sympathy with republican France, but in law and tradition, and in that deeper instinct which underlies both, as English as ourselves.

It is not so to-day. Even excluding the sprinkling of aborigines, and the negroes whom, for weal or woe, America has itself brought into the fold, you find in an American city such a mixture of races as perhaps has not been seen since the days of imperial Rome, that drew her citizens from the whole of a conquered world. We should have said her inhabitants; the proud *civis Romanus sum* was reserved for true-born Romans alone, save when the title was conferred as a grace to which a patent of nobility is inferior. But the American immigrant can, within a few years, become an American citizen, and influence in his degree the policy of a nation whose utmost future we cannot yet foresee. *Civis Americanus sum* is a title fraught with so great a possibility that we can fully justify the authorities in exercising the severest restrictions as to whom they admit—if only these restrictions are wise.

A recent American writer has complained that immigrants no longer come to the Western continent to share in American liberty, but to grasp some portion of American wealth. His contention might well be reversed. The American immigrant of fifty years ago left his home because he had heard of work and wages there which he could not obtain where he was. The reason was very rarely political, almost invariably it was a matter of more money for himself and better posibilities for his family. An examination of the nationalities that supplied the immigrants, as compiled by the official Bureau of Statistics at Washington, will make this clear. Ireland has always supplied a large proportion of immigrants, but it was not till after the great famine that the real rush of Irish emigration began. Between 1841 and 1850 we find over 780,000 Irish among the emigrants, and between 1851 and 1860, 914,000. The next decade shows 435,000, and that ending in, 1880 436,000. Then again a great increase is observable in the next ten years, when the total is 655,000. This may have been due partly to political causes, for then the Home Rule agitation was at its fiercest; but, in that case, how are we to explain that emigration from England reached its maximum at the same time, and even exceeded that from Ireland by 2,000 souls? Scotland also sent its largest number —150,000—though, as a large proportion of Scottish emigrants go to Canada, these figures give no accurate notion of the total amount of emigration from that country. But if those parts of the United Kingdom which had no political grievances of any magnitude went away in such large numbers the causes must have been chiefly economic.

The Irish immigration to the United States is, however, no longer the largest. Germany, which between 1820 and 1830 contributed only 6,761 to the alien population, has since that time sent annually increasing numbers. Within the next ten years the immigration reached 152,000; between 1841 and 1851 the number was 434,000, and the next ten years brought 951,000. At this date the German immigration exceeded the Irish, and has since kept and increased its pre-eminence. Between 1881 and 1890 the German immigrants reached the enormous number of nearly a million and a half, and the statistics of the three last years show that they are still pouring in at the rate of 113,000 per annum. Of these latter-day Germans a number, larger perhaps than the proportion of Irish, are self-exiled for political reasons. Many of them go to Illinois, to Chicago, that hotbed of all seething restless elements, and thence spring Anarchist plots, reckless murders, endless industrial troubles; but all the Teutons are not of these. Indeed, the Irishman and the German have each their special function in American life. The first betakes himself to politics, "as 'tis his nature to," or to some position in the public service obtained and maintained by political influence; the second quenches the thirst of the great Republic. An American music hall song is founded on these two characteristics. Hans and Patrick, crossing in the same steamer as steerage passengers, have a quarrel, in which the German has the best of it. The other vows to be avenged some day, and within a fortnight of their landing Pat, become a policeman through the influence of some of his countrymen, arrests Hans for selling beer without a licence.

It must be noted that Germans here mean ex-subjects of the German Empire. The first Austrian emigrants appear in the decade 1861 to 1870, to the modest number of 7,800. Since then they have come ever more rapidly, and within the last three years no fewer than 210,000 have settled on American shores. But another immigration seems to be increasing more quickly. Italy, which sent only 408 representatives between 1821 and 1830, has since 1890 sent 211,000. Not over-welcome are these Italian immigrants. Some of them make, it is true, good navvies, and they will, at least at the beginning of their career, work for moderate wages, a fact which wins them the enmity of the more exacting and extravagant native American; but, on the other hand, many of them are enrolled in the Mafia, or some kindred secret society, which uses the stiletto and the revolver as readily in New York or New Orleans as in Naples or Sicily.

Two nations are set down as having each added 91 souls to the American nation between 1821 and 1830, Scandinavia and Russia, including Poland. The Norwegian invasion has steadily gone on, every year showing a steady increase, till between 1881 and 1890 more than half a million came westward, and the rate of 50,000 a year has been steadily kept up since. They make good citizens, these men of the North, not quite so quick and active as the Yankee desires, and more moderate in their ambitions than he can yet conceive of being, but peaceful, industrious, and law-abiding. From Russia there came during fifty years only a few, and these chiefly exiled or fugitive Poles. But between 1871 and 1880, 52,000 landed. Then, within the next ten years, came a sudden rush, and the record stands at 265,000. Remarkable to say, or so it would be if one did not know the pitiable cause, the last three years show a total not far from being as large as that of the ten that went before, no fewer than 250,000 having come between 1891 and 1893. These are Russian Jews driven out by the religious zeal of the Czar, and for the most part totally unsuited to their new environment. They know no language but their own, their habits are insanitary to the last degree, and the industries they follow are among the poorest open to skilled labour. Yet, through the short-sighted self-distrust that characterises American policy, such as these are permitted entrance, while good artisans, if they are prudent enough to secure a position before they leave home are fined £200, as being "imported contract labour."

